# Effectiveness of a Teacher Training Program for Students with Symptoms of Developmental Disorders: Data from a Correspondence High School in Japan

**DOI:** 10.3390/ijerph17093100

**Published:** 2020-04-29

**Authors:** Atsuko Ishii, Hiroko Okuno, Takayoshi Nakaoka, Hidemi Iwasaka, Masako Taniike

**Affiliations:** 1 United Graduate School of Child Development, Osaka University, Osaka 565-0871, Japan; okuno.h@kokoro.med.osaka-u.ac.jp (H.O.); masako@kokoro.med.osaka-u.ac.jp (M.T.); 2 Clark Memorial International High School, Hyogo 669-1512, Japan; t.nakaoka@clark.ed.jp; 3 Developmental Center for Child and Adult, Shigisan Hospital, Nara 636-0815, Japan; iwasaka@heartland.or.jp

**Keywords:** teacher training, adolescents, developmental disorders, teacher’s report form, social responsiveness scale-2, teacher’s confidence

## Abstract

In the present study, a teacher training program based on behavioral therapy was conducted for high school correspondence course teachers of adolescents aged between 15 and 18 years who showed developmental difficulties. Participating teachers were assigned to either an immediate treatment (IT; *n* = 13) or delayed treatment control (DTC; *n* = 17) group to evaluate the effectiveness of the program, which comprised five 90-min sessions with small groups of three to six participants and was conducted over three months. The results showed significant improvement in students’ behaviors and social responsiveness and in teachers’ confidence among those in the IT group; however, those in the DTC group did not show any such improvement. We discuss the program’s feasibility in terms of developing support resources for teachers in Japanese high schools.

## 1. Introduction

Correspondence high schools have played a key role in offering education to working youths since the end of World War II in Japan, which was their original purpose [1]. However, the occupational status of correspondence high school students has changed, and the percentage of full-time employed students has decreased from more than 60% in 1982 and approximately 30% in 1994 to 2% in 2016 [2]. According to a report on correspondence high schools in Japan, the top two reasons students choose a correspondence high school are high school graduation requirements for most professions (45%) and the freedom to learn at their own pace (17%), while a much less-common reason is work demands (5%) [3].

This system requires remote studying, submitting reports, direct schooling, and taking examinations [1]. Students generally take courses through remote learning with the option of a computer-based home-school program; therefore, they have less direct schooling time with teachers than full-time and night-school students do. However, to further support students’ learning, correspondence courses of different frequencies have been offered, such as a five-day schooling course or a once-a-month schooling course [1].

Another change made over time is an increased number of students. Owing to the decreasing birthrate in Japan, the number of full-time and night high school students has been decreasing since 1989; in contrast, the ratio of correspondence high school students to all high school students has been increasing since 2004 [1]. In addition, the number of students enrolled in private correspondence high schools exceeded that of public correspondence high schools in 2007 [1], and in 2018, private correspondence high schools had more than twice as many students as public ones in Japan [2].

Currently, students choose correspondence high schools for various reasons, such as having less experience going to school or dropping out from other high schools [1]. Furthermore, an increasing number of students with mental health disorders, including developmental disorders (DDs), attend correspondence high schools [4]. According to a report by the Ministry of Education, Culture, Sports, Science, and Technology (MEXT) [5], 2.2% of all high school students in Japan have DDs, such as autism spectrum disorder (ASD), attention-deficit hyperactivity disorder (ADHD), or a learning disorder (LD). However, correspondence high schools have the highest percentage of such students, with 15.7% (1.8% in full-time high schools and 14.1% in night high schools). Another report by the MEXT [6] stated that support systems for kindergarten and high school students with DDs have not improved, compared to those for elementary and junior high school students.

In correspondence high schools, teachers often face difficulties in dealing with such students, including those identified in one study as the interpersonal scene, learning guidance and evaluation, and home environment and parents’ assistance. Further, teachers have reported finding it difficult to support students who are absent for a long time and different types of students having DDs or other mental health disorders [7]. Adolescents displaying symptoms of DDs have difficulties in relationships with both peers and adults, owing to their behavioral problems and poor academic performance; among adolescents with ADHD, there is also a lack of emotional control [8]. Additionally, core symptoms of adolescents with ASD, such as difficulties in reciprocal interactions and communication and restricted interests or repetitive behaviors, are difficult for teachers to handle [9]. Correspondence high school teachers are expected to support these students and their parents both psychologically and practically; however, in Japanese high schools, support systems and aids for students with DD symptoms are underdeveloped. In 2007, the MEXT established some model high schools across Japan to implement and develop support systems for students with DDs [10], and they initiated a new system of special support services in resource rooms at high schools in 2018. However, high school teachers still need to learn special educational methods for students with DDs [10]. Sekine [11] conducted a survey of correspondence high school teachers and stated that it is difficult for teachers to deal with individual students carefully enough because of too many students to be taken care of per one teacher and that students could receive more suitable guidance at special education schools. Correspondence high school teachers usually do not receive special education training; however, they have the highest percentage of students with developmental difficulties in Japanese high schools. Thus, there is room for improvement in teacher training (TT) systems and backup support for teachers. The diverse support needs required by students at correspondence high schools burden teachers and decrease their confidence in dealing with such students. Adolescence challenges students with DDs and their supporters because developmental tasks, such as identity or independent tasks, are abstract and individually different [12]. In addition, special support for students with DDs is crucial not only to promote students’ quality of life but also to ensure they graduate from high school and bridge the gap in support to their next step after graduation [10].

In the Japanese educational system, a few TT programs have been implemented for teachers who support children with DDs at nursery and elementary schools [13,14,15]. These TTs are based on UCLA’s parent training (PT), originally developed for families of children with ADHD [16,17], and modified for the Japanese population [18]. This TT program aims to enable teachers, who observe and record students’ behaviors at school, to alter their own behavior toward the students and enhance their confidence through positive interactions with them [14,19]. Both in Japan and abroad, TT for those who teach young children has been effective [14,20,21,22]. The teachers develop more positive and inclusive attitudes toward students because they increase their experience and knowledge about students’ difficulties [23] and develop skills to deal with students’ maladaptive behaviors [14].

However, to date, no study has examined the effectiveness of TT for correspondence high school teachers. There has been little improvement in the support systems available for adolescents with DDs attending high school, as the TT program was exclusively developed for younger children. The percentage of students with DDs attending correspondence high schools is much higher than full-time high schools [5], which creates a need for an effective support system for them and their teachers. Therefore, the present study introduced a TT program for teachers of adolescents showing DD tendencies at X correspondence high school and evaluated its effectiveness on students’ behaviors and social responsiveness, as well as teachers’ confidence in dealing with such students.

## 2. Materials and Methods

### 2.1. Institution Setting

X correspondence high school is a private high school with campuses across Japan. Japanese correspondence high schools generally offer courses to students through remote learning with options of computer-based home-school programs, main and elective classes, and credit-recovery support. X offers different types of schooling programs, including home-study, weekend, and weekday courses (e.g., a five-day course). Providing more direct schooling days, such as a five-day course, is a current trend in Japanese correspondence high schools because longer schooling time provides more opportunities for students to complete mandatory reports [1]. In this TT program, the participating teachers were in charge of weekday course students and met the students Monday through Friday, from morning to evening. X has multiple campuses across Japan, and teachers deliver educational services using a standard curriculum (i.e., same teaching materials and methods), which was developed by school administrators.

### 2.2. Participants

Thirty-five (24 men and 11 women, aged between 22 and 59 years) high school teachers of students with tendencies toward or showing symptoms of DDs at X correspondence high school applied for this intervention program.

The inclusion criteria for the participating teachers were (1) working full-time at one of X’s campuses, (2) being in charge of a target student with (or showing signs of) DD, and (3) having issues dealing with said student. As this research program was a teacher–student dyad intervention, the exclusion criteria for the participating teachers were (1) the paired target student’s inclusion criteria were not met or (2) the student was excluded.

The inclusion criteria for the target students were (1) fall within the assistance-need areas of the Adaptation Scale for School Environment on Six Spheres [24]; (2) have at least one higher score than the cut-off on the Checklist for LD, ADHD, and high-functioning autism [25]; (3) have a higher total score than the cut-off for clinical borderline behavior on the Japanese version of the Teacher’s Report Form (TRF) [26]; (4) belong to a weekday course (Monday to Friday, from morning to evening, at X correspondence high school); (5) be aged 15–18 years. Additionally, students who (1) had been absent from school for more than 30 days, (2) had been taking medication and changed their dosage during the program, or (3) underwent another program within three months of the beginning of this research, such as social skills training, were excluded from this study.

In the control condition, three students did not meet the third inclusion criterion and two other students met the first exclusion criterion; therefore, the data from 30 participating teacher–student dyads (teachers: 23 men and 7 women, aged between 22 and 56 years) were analyzed after they were assigned to either an immediate treatment (IT) group (*n* = 13; 9 men and 4 women) or a delayed treatment control (DTC) group (*n* = 17; 14 men and 3 women), according to their school schedules. Before starting the first intervention session, an intake interview was administered individually with each participating teacher by the first and third authors. All the teachers had issues supporting the paired target student, and they were asked to discuss the most difficult issue(s) using a free-response method.

Table 1 shows the results of teachers’ answers in percentages. Examples of major issues included supporting the target student’s academic skills; reducing the student’s maladaptive behaviors toward teachers, such as constantly seeking the teacher’s attention; reducing troubles with peers; dealing with the student’s panic or emotional problems; and ensuring that the student understands the teacher’s instructions.

The target students (*n* = 30; 25 boys and 5 girls) were placed into either an IT (*n* = 13; 11 boys and 2 girls) or DTC (*n* = 17; 14 boys and 3 girls) group, in accordance with their paired teacher. According to the target students’ LD, ADHD, and high-functioning autism scores [25], assessed by teachers and used as an inclusion criterion, most target students scored above the cut-off points on two or three categories (Table 2). In contrast, fewer students obtained scores over a single cut-off point (Table 2). The demographic information of participating teachers and students is presented in Table 3.

### 2.3. Measures

The questionnaires described below were used to evaluate the effectiveness of this study by examining students’ behaviors/social responsiveness and teachers’ confidence in dealing with such students pre- (baseline; Time 1) and post-intervention (Time 2). Semi-structured interviews were also conducted to ask participating teachers about their satisfaction with this program after each TT intervention (at Time 2 for the IT group, and at Time 3 after the DTC intervention period for the DTC group).

#### 2.3.1. Teacher’s Report Form of the Child Behavior Checklist (TRF)

The TRF [26] is a 113-item, teacher or educational professional-report scale used to rate children and adolescents aged between 5 and 18 years. It determines the most frequent behaviors or emotional problems observed in the school environment. The original TRF (in English) has been translated into Japanese with high construct and concurrent validity [27]. It is measured on a three-point scale and has eight subscales: withdrawn/depressed, somatic complaints, anxious/depressed, social problems, thought problems, attention problems, delinquent behavior, and aggressive behavior [26]. The Japanese version of the TRF has high internal consistency for all scales [28]. The participating teachers scored the paired students using this scale.

#### 2.3.2. Social Responsiveness Scale-2 (SRS-2)

The SRS-2 [29] is a 65-item, parent- or teacher-report scale used to rate children and adolescents aged between 4 and 18 years that measures levels of autistic behaviors in daily social settings. The SRS-2 has been translated into Japanese with high construct and concurrent validity, and the Japanese version of the SRS-2 also has good psychometric properties [30]. It is measured on a four-point scale and has five subscales: social awareness, social cognition, social communication, social motivation, and restricted interests and repetitive behavior (RRB). The participating teachers scored the paired students using this scale.

#### 2.3.3. Confidence Degree Questionnaire (CDQ) for Teachers

The CDQ for Teachers is a modified version of the CDQ for Families [18], which measures parents’ confidence in undertaking childcare under PT programs. Wording for some of the items was modified, as follows: (Q8) from appropriate support and communication in the “family” to “in school”, (Q12) from reducing troubles “at home” to “at school”, (Q13) and (Q14) from “other family members” to “other teachers/coworkers”, and (Q15) from “other families” to “other teachers”. The CDQ for Teachers is measured with a five-point scale and assesses teachers’ confidence in supporting students at school. The modification of the teacher version was approved by the fourth author, who originally devised the CDQ for Families [18]. The difference between the CDQ for Families and the CDQ for Teachers is that the latter excludes two questions on medications because parents take care of this at home. The CDQ for Families has not yet been standardized; however, it has been used to assess parents’ confidence about the childcare they provide in PT programs in Japan [31]. Therefore, only the individual questions, and not the total score, were analyzed. Data were compared with previously reported results [31]. The participating teachers scored themselves using this scale.

#### 2.3.4. Semi-Structured Post-Program Interviews

After the TT program intervention (at Time 2 for the IT group and Time 3 for the DTC group), semi-structured post-program interviews with each participating teacher were conducted by the second author to ask teachers about their satisfaction with this program, changes in their paired student’s behavior, and changes in their own cognition and behavior. The qualitative answers from semi-structured interviews were categorized by two researchers for the analysis to pursue the main themes in the interview and explore them from a new angle, by grouping the information and labeling the category groups. The KJ method was used [32], which is a qualitative analysis method in Japan. These qualitative data were collected to complement the findings from quantitative measures.

### 2.4. Procedures

This research received ethical approval from the Ethical Committee of Osaka University Hospital (no. 16535-6). After approval, a research plan was presented for campus principals at X correspondence high school, and the high school agreed to place recruitment brochures for teachers at campuses and have recruitment talks with prospective participating teachers over two weeks. During the recruitment period, 35 teachers applied for the program. Each participating teacher listed one paired target student with DD tendencies from among their own weekday course students. The matching of teacher–student dyads was done by each participating teacher when he or she applied for recruitment. Based on participants’ inclusion and exclusion criteria, five pairs were excluded, as mentioned above, and the number of participating teachers became 30.

All participants provided written informed consent, and the TT intervention program was conducted from September 2017 to March 2018. To compare the groups’ performances during the first three months, the IT group underwent the TT program, and in the latter three months, the DTC group underwent the program. This TT program was designed to have three to six fixed participating members in one group; therefore, both the IT and DTC group participants were divided into four small groups each; for example, Monday, Tuesday, Wednesday, and Thursday groups, according to teachers’ schedules. The whole program spanned three months, with sessions every other week. Overall, five 90-min sessions were conducted, starting at 5 p.m. (after the teachers’ workday). After the Time 1 assessment, the DTC group teachers had no contact with the research members until the Time 2 assessment. The IT and DTC group teachers were never in the same session groups.

The attendance rate of teachers for this program was 95.38% in both groups. Additionally, no participants dropped out of either group during each intervention period. However, during the waiting period, three of the DTC group participants declined to undergo the program because of changes in their job schedules at the beginning of the DTC group intervention.

All treatment sessions were facilitated by the first author, a licensed school psychologist, and a doctoral student researching child development. The first author joined two cycles of a PT program [31] as a sub-facilitator at Osaka University Hospital and had received supervision by the trainer. To maintain program fidelity, all TT sessions were recorded, so the supervisor could check whether all program content was covered and if the facilitating methods were appropriately used to manage sessions, using a check sheet. Ten scores were given in 10 categories per session; for instance, clear lecture skills in detail, appropriate feedback on homework, and clear advice depending on a target students’ difficulties [33]. The score was evaluated by the supervisor for all the sessions and calculated as the average percentage of achievement. The assessment indicated an average accuracy of 97% for the program managed by the facilitator.

#### 2.4.1. TT Program

The current research utilized a Japanese version of the TT program [13,14,15]. The program was intended for teachers of younger children with DDs; therefore, some modification was needed to make the program suitable for teachers of adolescents [34,35,36,37].

Before the program intervention (at Time 1), intake interviews were conducted with all the participating teachers from both the IT and DTC groups by the first and third authors to list their major issues with each target student, problems, and goals that needed to be set to support the students (Table 1). At Session 1, participating teachers confirmed their goals and were trained in core methods of behavior therapy by observing the target students’ behaviors [33]. The current TT program taught teachers to divide student behaviors into the three categories of appropriate, not-so-appropriate, and inappropriate behaviors to stop, with the aim of increasing appropriate behaviors and reducing the latter two behaviors [33]. Session 2 focused on reinforcing students’ desirable behaviors by giving them positive attention. As one modification method for adolescents, participants were trained in using tailored and “I-message” praising [37]. Other important modified tools were addressed in Session 3. Regarding clear instruction skills, a focus was placed on the importance of supporting adolescents’ pre-academic skills. Participants were trained in providing concrete motivational support to improve students’ skills, such as managing homework and note-taking [35,36]. The content of the whole program is shown in Table 4.

Each session required teachers to complete a homework report on the previous content and comprised a lecture, role-playing scenario, and announcement of the homework to follow. Between sessions, teachers tried using methods from the program with each target student at school and brought the records as homework to share with other participants in the next training session. The program aimed to include a comfortable discussion group for teachers with fixed program members, who had similar difficulties in supporting target students, and those activities were used as situations from which all participants learned from their peers [33].

#### 2.4.2. Treatment Integrity and Ethical Considerations

The fidelity of the program was managed by the supervisor, as mentioned above. The personal information of the participants was protected, following the protocol approved by the ethical committee at Osaka University Hospital. Thus, the treatment integrity and ethical considerations of this research were ensured.

### 2.5. Data Analysis

The data were statistically analyzed using SPSS Statistics Version 25 (IBM Corp, Armonk, NY, USA). First, a series of baseline analyses was conducted to ensure that the groups had homogeneity pre-test. Then, an analysis of covariance (ANCOVA) was performed to control differences between groups at Time 1. To apply the ANCOVA, assumptions of normality, homogeneity of variance, homogeneity of the regression slope, and the reliable measurement of the covariate were checked, and only the data that met all the assumptions above were considered for discussion. Independent variables were time (pre = Time 1 vs. post = Time 2) and group (IT vs. DTC). Effect sizes are shown with partial eta squared.

## 3. Results

### 3.1. Baseline Results

According to the results of the *t*-tests, Wilcoxon rank-sum tests, and chi-square analyses, no significant differences were found at baseline regarding demographic characteristics of participating teachers and target students in the IT and DTC groups. Table 3 shows that, among teachers at baseline, both groups did not significantly differ in age, current career, sex, or educational degrees. Among students at baseline, the groups did not significantly differ in age, grade, sex, LD, ADHD, and high-functioning autism scores, or diagnosed rate. However, for baseline scores of the following subscales, *t*-tests and Wilcoxon rank-sum tests for analyzing group differences among the dependent variables revealed that there were significant differences in communication and motivation for SRS-2 and for two questions (Q14 and Q15) of the CDQ for Teachers between these two groups.

### 3.2. Intervention Results

#### 3.2.1. Teacher’s Report Form of the Child Behavior Checklist (TRF)

There was a significant difference between the total scores of the IT and DTC groups [F(1, 27) = 11.04, *p* = 0.003, partial *η*^2^ = 0.29] (Table 4). On the subscales for social problems, attention problems, and delinquent behavior, the IT group showed significant improvements [F(1, 27) = 7.40, *p* = 0.011, partial *η*^2^ = 0.22; F(1, 27) = 5.07, *p* = 0.033, partial *η*^2^ = 0.16; and F(1, 27) = 6.55, *p* = 0.016, partial *η*^2^ = 0.20, respectively] (Table 5).

#### 3.2.2. Social Responsiveness Scale-2 (SRS-2)

The total score of the IT group only revealed a tendency for improvement compared to the DTC group (*p* = 0.050). The subscale of restricted interests and repetitive behavior (RRB), however, showed a significant improvement in the IT group compared to the DTC group [F(1, 27) = 4.38, *p* = 0.046, partial *η*^2^ = 0.14] (Table 5).

#### 3.2.3. Confidence Degree Questionnaire (CDQ) for Teachers

We observed that the items Q1 “waiting for the student’s growth” and Q2 “accepting the student’s developmental difficulties” significantly improved in the IT group [F(1, 27) = 7.60, *p* = 0.010, partial *η*^2^ = 0.22, and F (1, 27) = 5.81, *p* = 0.023, partial *η*^2^ = 0.18, respectively]. Additionally, three other items Q5 “making a space for the student to relax”, Q7 “dealing with the student’s maladaptive behaviors”, and Q12 “reducing troubles at school because of the student’s behaviors” showed more significant improvement in the IT group than in the DTC group [F(1, 27) = 8.29, *p* = 0.008, partial *η*^2^ = 0.24; F(1, 27) = 7.96, *p* = 0.009, partial *η*^2^ = 0.23; and F(1, 27) = 12.63, *p* = 0.001, partial *η*^2^ = 0.32, respectively] (Table 5).

#### 3.2.4. Semi-Structured Post-Program Interviews

The semi-structured post-program interviews of all the participating teachers recorded opinions on their satisfaction with this program and modifications or changes of the target student’s behaviors, as well as to their own cognition and attitude toward the students. Answers from the semi-structured interviews were transcribed by the second author and categorized by two researchers to extract quantitative data using the KJ qualitative analysis method [32]. As Table 6 shows, 88.46% of the total IT and DTC group teachers were satisfied with the program, 80.77% observed modifications in their students’ behaviors, and 92.31% answered that their cognition or attitude toward the target students had changed after the program intervention.

## 4. Discussion

### 4.1. Changes in Teacher’s Report Form of the Child Behavior Checklist (TRF) Scores: on Student Behaviors

The total TRF score of the IT group improved significantly, and the subscales of attention problems, delinquent behaviors, and social problems showed significant improvements in this group (Table 5). According to previous research, a PT program for parents of adolescents with DDs [38] resulted in improvements in attention problems, and a TT program for teachers working in elementary schools reduced children’s symptoms of ASD that disrupted the class [39], as well as improved children’s social skills [40]. The current study’s findings are consistent with those of previous research. One possible reason could be that, as Table 4 shows, because the program was adjusted to fit adolescents’ developmental features, the current TT enabled the intervention to support executive functioning skills, such as pre-academic and autonomous management skills, with appropriate motivations, which Sibley and her colleagues emphasized [41].

First, TT content on tailored support for students’ pre-academic skills enabled improvements in student’s attention abilities. As shown in Table 4, “clear instructions” in Session 3 included not only the teacher’s instructions through multisensory support tools and ordering tasks to help students follow the lesson [42] but also their support of students’ pre-academic skills, such as organizing and homework skills [43,44]. With concrete skill-building support from teachers, including multisensory methods, we believe students could show improved attention in school.

In addition, Session 3 of the TT program involved supporting students in developing autonomous management skills, such as document management, which can enable students to improve their self-management sufficiency [35], a necessary skill for adolescents, which we believe complemented their attention abilities. The current research findings imply that successful behavior modification for adolescents requires a strategy for students to acquire self-management skills [36]. Our program trained teachers to support adolescents in improving their pre-academic skills, such as enhancing autonomous self-management skills [45] during class or break time at school, which enabled students to concentrate more in class and led to reduced attention problems.

Second, we believe students’ maladaptive behaviors improved due to the modified relationship between the teacher and target student, which resulted from training teachers to show appropriate reactions toward students’ appropriate behaviors, such as by “praising students immediately after an appropriate behavior” [18]. This was included in Session 2 of the TT. In particular, training teachers on how to adjust their praising methods for adolescents (see Table 4) allowed for better communication between the teacher and the target student [37]. In addition, the ignoring technique to deal with students’ maladaptive behaviors could be used in a healthy relationship environment, which we think further encourages students to follow the rules and instructions, thereby reducing behavioral problems. These praising and ignoring techniques enabled the creation of an “environment that maximizes their strengths while minimizing the influence of deficits” [35] (p. 10), which is crucial for adolescent support. A key developmental aspect for adolescents is protecting their pride, especially by not humiliating them by focusing on their deficits in front of peers [37], which is important and enhances self-esteem. Therefore, the environment created by teachers with a more positive focus on student advantages worked effectively. Moreover, because adolescents build new relationships with adults outside of their family after becoming mentally independent from their parents [46], positive evaluation from school teachers is an effective support for high school students. In other words, such an approach adopted by teachers could provide target students with a chance to attain positive attention, thus reducing their maladaptive behaviors.

Third, the improvement of social problems was promoted within the inclusive environment of X correspondence high school. Lack of assistance from teachers and exclusion from the classroom can aggravate students’ social and academic problems [47]. However, at the X high school campuses, teachers assisted each target student with tailored interventions and support methods within an inclusive environment to reduce social problems. Interventions for adolescents do not rely on managing a perfect environment because of the independent features of adolescent development tasks [48]. The intervention environment of the current research was appropriate and feasible, as teachers could assist target students by imparting self-reliance skills under such circumstances. Furthermore, because of the inclusive setting—for example, when a teacher praised the target student’s good behavior using the praising technique of Session 2 (Table 4)—teachers also praised the whole class, as the other students behaved appropriately at the same time. This technique highlights an important point based on the universal design of enhancing all students’ skills, as teachers deal with target students and the group simultaneously. Teachers’ inclusive attitudes can be a factor that influences the formation of an inclusive atmosphere for peers to be more accepting of students with DDs in their classrooms [49]. This, we believe, could work to reduce target students’ social problems.

### 4.2. Changes in Social Responsiveness Scale-2 (SRS-2) : on Student Social Responsiveness

For the SRS−2, an improvement on the subscale for restricted interests and repetitive behavior (RRB) can be attributed to the fact that environmental accommodation depending on each need [50] created a situation in which tendencies of RRB were lessened, rather than completely disappeared. According to Boulter and colleagues’ anxiety model of ASD, the three factors of social/environmental issues, rigidity of thought and difficulty with emotion processing, and sensory sensitivities led to RRB and anxiety via intolerance of uncertainty [51]. The TT program trained teachers to set up environmental accommodations, such as preparing relaxing spaces and predictable plans adjusted to individual student support needs and addressing sensory issues (Table 4). By undergoing the TT program, teachers could acquire the necessary knowledge to affect cognitive changes in students with DDs [22] and appropriate support methods to deal with RRBs. For example, having structured environmental settings in classrooms, such as seating arrangements, consideration of sensory issues, or showing prospective plans, is known to cause fewer restricted and repetitive tendencies with fewer sensory stimuli [50]. As a result, RRBs were considered to be improved in the current study.

Additionally, as Boulter and colleagues’ anxiety model [51] shows, students’ emotional control skills in TT Session 5 (Table 4) could contribute to the reduction of RRBs. Other factors could be modified teacher–student relationships and praise-and-praised relationships, as mentioned above.

### 4.3. Changes in Confidence Degree Questionnaire (CDQ) for Teachers: on Teacher Confidence

Regarding degrees of teacher confidence, the following five items showed significant improvement: (Q1) “waiting for the student’s growth”, (Q2) “accepting the student’s difficulties”, (Q5) “making a space for the student to relax”, (Q7) “dealing with the student’s maladaptive behaviors”, and (Q12) “reducing troubles at school because of the student’s behaviors”. Compared with previous research results on parent confidence from PT [31], the impact of improvement of CDQ for teachers was smaller. The number of items that showed significant improvement in this study was about half of that in the previous research, and all five items also showed significant improvement in previous research findings [31].

Regarding Q1 “waiting for the student’s growth” and Q2 “accepting the student’s difficulties”, as Table 6 shows for changes in teachers’ cognition or attitude (“Did you find any changes in your cognition or attitude toward the target student?”), 92.31% of teachers complementarily indicated such changes. Having detailed knowledge and support methods for students with DDs changed teachers’ cognition [22] and enabled them to accept students’ difficulties through behavioral intervention [20,52]. Like Fukuda and colleagues argued, by thoroughly observing each target student, teachers can deepen their interpretation of target students’ tendencies and accept their difficulties [20].

Regarding Q5 “making a space for the student to relax” having something to do with the improvement of restricted interests and repetitive behavior (RRB) on SRS-2, Session 3 of TT, environmental accommodations (Table 4) created a situation in which fewer RRBs appeared, as discussed above, and teachers could recognize the importance of environmental settings in making a space for students with DDs to relax. Another reason could be teachers’ keen observation skills, which helped increase their competence in setting up spatial structures [39] before their target students experienced panic. Table 6 also indicates the complementary support of this improvement on Q5, in which 60.00% of teachers were able to refine their target students’ emotional control.

Regarding Q7 “dealing with the student’s maladaptive behaviors”, previous TT research [14,20] revealed that behavioral analysis enables nursery and elementary school teachers to react appropriately to and modify children’s maladaptive behaviors. Behavioral modification in the current TT intervention was based on dividing student behaviors into three categories of (1) appropriate, (2) not-so-appropriate, and (3) inappropriate behaviors to stop, with the aim of increasing appropriate and reducing inappropriate behaviors [49] (see Session 1, Table 4). The results in the current study for the CDQ for Teachers on handling students’ inappropriate behaviors were consistent with the findings of Onishi and colleagues [14]. Teachers’ interview results (Table 5) also showed increased confidence in dealing with students’ maladaptive behaviors and identifying more appropriate behaviors at school. Of the major issues, (1) showed that 75.00% of teachers reported the target student developed an appropriate studying attitude and pre-academic skills, and (5) showed that 62.50% reported students changed their behavior in order to follow teachers’ instructions.

Lastly, regarding Q12 “reducing troubles at school because of the student’s behaviors”, common discussions on improvements in maladaptive behaviors and social problems of TRF and RRB of SRS-2 mentioned above concern teachers’ increased confidence in reducing students’ troubles at school. With modifications in students’ actions related to maladaptive behaviors, social problems, and RRBs, teachers recovered their confidence in reducing students’ troubles. Although there is a limitation in terms of the lack of correlation analyses reflecting this change, teachers’ comments in the interviews, as shown in Table 6, complementarily indicated the relationship; 91.67% of teachers noted students’ improvements in behaviors toward teachers (Major Issue 2), 60.00% on emotional control (Major Issue 4), 71.43% on rule-breaking (Major Issue 7), and 60.00% on reducing impulsive or hyperactive behaviors (Major Issue 8). In contrast, lower numbers were reported for behaviors toward peers (30.77%, Major Issue 3) and friendship with peers (12.50%; Major Issue 6). According to results from teachers’ interviews, students showed a reduction in maladaptive behaviors toward teachers of 90%. However, only maladaptive behaviors toward peers only reduced by 30%. The greater difficulty in target students’ relationship-building with peers rather than adults, such as teachers, is inherent in the psychological development process of adolescents with DDs [53], which is consistent with the results of the current study.

### 4.4. Implications

These findings highlight the feasibility of introducing TT to teachers of adolescent students who present symptoms of DDs and have difficulties at correspondence high schools in Japan. Correspondence high schools have diverse students, including students with typical developmental needs and students with DD-based support needs. To effectively assist with the needs of adolescent students, supporting teachers’ difficulties are urgently needed. Further, our findings suggest that the TT program enables teachers to learn individually tailored support methods through regular group discussions with other teachers who have similar worries and stressors, even for students with different symptoms. We contend that TT for teachers of adolescents should include a basic behavioral therapy method to cover different types of DDs, so that the facilitators can tailor their advice and support to the participating teachers, depending on their students’ major issues and difficulties.

Further, the intervention environment of the current research was a semi-structured school environment, which lacked an environmental structure adjusted for each student’s needs, such as at special support education schools. Regarding support for adolescents, perfect structural adjustments at school are difficult due to the development and complications of interpersonal relationships [54]. The current research could utilize such a semi-structured environment in which teachers could adjust individual supports or environmental settings for adolescents with either DDs or typical development. Therefore, the feasibility of introducing this TT program to correspondence high school teachers within a semi-structured setting was shown by setting goals for individual target students and holding discussions on individual support needs in each session of the program. Especially at the diverse sites of the correspondence high school, which have students with different support needs who display symptoms of DDs, we believe that an individual support system like that of the present program would work effectively as a simple support method.

### 4.5. Limitations

Despite the study’s contributions to strengthening the teachers’ skills in correspondence high schools in Japan, certain limitations existed with respect to the sample size and generalizability. The teachers who participated in this program were those who were personally motivated to do so; thus, the sample comprised only some teachers working at a private correspondence high school. The target students paired with each teacher were limited to those in the weekday courses because students at correspondence high schools urgently need appropriate daily support from teachers who can spend enough time assisting them Monday through Friday, morning to evening. However, in addition to the weekday courses, correspondence high schools also have students who take weekend and home-study courses, who study at their own pace at home and spend less time at school. Correspondence school students with DDs can have a lot of difficulties, including extending the period of absence from necessary schooling, dropping class credits, or dropping out of school entirely [1]. We were unable to assist students who attended courses with fewer schooling days. Moreover, the sample of target students was likely biased by the teachers choosing each target student, whose problems may have been allayed through this program. In terms of sample size, we could not obtain large effect sizes due to the small numbers of participants. Thus, a replication of the study with a large sample size, different population, or a more exclusive focus on specific types of student difficulties and schooling courses would be useful to test the generalizability of our findings.

Another limitation is that this was not a researcher-blind study. The first author facilitated the program and analyzed the data. Even with data anonymity using IDs and data collection by other researchers who did not facilitate the program, we cannot deny the slight possibility of researcher bias. Moreover, as the questionnaires were completed by the participating teachers, future research should use a more objective means of measuring data. A blind report by other psychological staff, or an evaluation by students—which takes more time to be understood in a school environment, considering the ethical issues related to personal information in Japan—could be undertaken in the future.

There are also some limitations pertaining to methodological requirements. The study was conducted during the last two academic trimesters because the teachers would have more accurate student information by the end of the first trimester. Further, the first trimester challenges students with symptoms of DDs due to the new class environment, teachers, and peers. The results of student behavior modification may have been different if the TT program was conducted during this period. We believe a trial performed during the first trimester might be helpful if the research goal were to focus on effective support at the beginning of a new environment. We also understand that it is necessary to conduct more longitudinal research, including a follow-up study, in order to evaluate the persistence of improvements through the duration of this program. Finally, our study design was quasi-experimental; thus, in the future, we would like to conduct a randomized controlled trial.

## 5. Conclusions

The current research introduced a TT program to high school teachers who are in charge of students with DDs or show DD symptoms at campuses of X correspondence high school. The findings supported the program’s effectiveness, showing improvements in students’ behaviors and social responsiveness and teachers’ confidence in dealing with such students. The difficulties and problems of adolescents with DDs might be considered too complex for their teachers to provide appropriate support because of the secondary disorders often arising from these difficulties. The implementation of TT, however, is imperative to support adolescents with DDs, specifically for teachers to build a positively focused psychological environment. This would reduce the antecedent conditions leading to students experiencing disadvantages in school, and provide a structure to facilitate this change. Currently, in Japan, correspondence high schools have more important roles in supporting students with different DD needs or tendencies in relation to the increasing number of such high school students. Due to this trend, the current study, with certain effective results, reported important indicators for further development of support programs for teachers who are in charge of students with DD-based support needs. Moreover, the support systems and tools for Japanese high school students with DDs are currently in development; thus, the practical feasibility of this program paves the way for further research on different types of educational environments, such as full-time and evening schools, not only correspondence high schools. Moreover, our research could further be extended to countries other than Japan.

## Figures and Tables

**Table 1:** Major issues of participating teachers (*n* = 30).

Content of Major Issues	*n* (%)
(1) Attitude toward studying and pre-academic skills: having the target student concentrate in class, take notes, meet assignment deadlines	18 (60.00%)
(2) Behaviors toward teachers: reducing the target student’s maladaptive behaviors toward the participating teacher	14 (46.67%)
(3) Behaviors toward peers: reducing interpersonal relationship problems among the target student and peers	14 (46.67%)
(4) Emotional control: dealing with the target student’s panic attacks or emotional control problems	10 (33.33%)
(5) Following the teacher’s instructions: having the target student follow the participating teacher’s instructions	10 (33.33%)
(6) Friendship with peers: having the target student build friendships because he or she has few or no friends	9 (30.00%)
(7) Rule-breaking: Reducing the target student’s delinquent behaviors	7 (23.33%)
(8) Reducing impulsive or hyperactive behaviors: reducing the target student’s aggressive or/and impulsive behaviors	5 (16.67%)
(9) Other	4 (13.33%)

Two participating teachers had a single major issue, while the others had two or more issues related to the target student with whom they were paired.

**Table 2:** Target students’ difficult tendencies related to developmental disorders ^1.^

Categories	LD, ADHD, and ASD	LD and ADHD	ADHD and ASD	LD	ADHD	ASD
IT (*n* = 13)	3	3	3	1	0	3
DTC (*n* = 17)	2	4	5	1	1	4

^1^ The number of target students with higher point(s) than the cut-off in each category [25]. IT: immediate treatment group. DTC: delayed treatment control group. LD: learning disorder. ADHD: attention-deficit hyperactivity disorder. ASD: autism spectrum disorder.

**Table 3:** Mean demographic and baseline variables for the immediate treatment and delayed treatment control groups.

Variable	Group				*p*
	Immediate Treatment		Delayed Treatment Control		
	*n* = 13		*n* = 17		
**Teachers**	M	SD	M	SD	
Age	29.15	7.18	34.76	9.48	ns
Current Career	2.50	3.93	3.38	3.84	ns
Men (%)	69.23		82.35		ns
Master’s degree (%)	7.69		5.88		ns
**Students**					
Age	16.00	1.00	16.65	1.00	ns
Grade	1.46	0.78	1.94	0.75	ns
Men (%)	84.62		82.35		ns
Checklist ^1^					
LD Listening	6.46	4.81	6.82	4.04	ns
LD Speaking	7.54	5.17	6.59	4.95	ns
LD Reading	3.31	4.40	5.29	3.89	ns
LD Writing	4.92	5.36	4.35	4.29	ns
LD Calculating	4.00	5.63	5.06	5.86	ns
LD Inferring	5.31	4.64	7.41	5.01	ns
Inattention	5.00	2.31	5.35	2.67	ns
Hyperactivity/Impulsiveness	2.85	2.94	2.35	2.74	ns
High-functioning Autism	27.08	13.07	20.53	11.64	ns
Diagnosed with DD (%)	46.15		23.53		ns

^1^ Checklist: checklist for LD, ADHD, and high-functioning autism [25]. M = mean; SD = standard deviation. LD: learning disorder. ns: no significant difference.

**Table 4:** Teacher training program content [38].

	Topic	Content Details	Homework
Session 1	• What are DDs?	• Observation of DDs and behavior therapy	• Observing S’s behaviors
	• Observing and understanding S’s Behaviors	• Problem setting: going over major issues about target Ss by teachers from the intake interview	
			• Dividing S’s behaviors into the three categories
		• Observing S from the target S’s eye line	
		• Dividing S’s behaviors into three categories: appropriate, not-so-appropriate, inappropriate	
Session 2	• Giving positive attention to the S’s behaviors	• Modified praising for adolescents; tailored praising, “I-message” praising	• Using praising skills
		• Effective praising in school settings: praising target Ss as well as the class in an inclusive environment	
Session 3	• Providing clear instructions and environmental settings	• Giving simple and clear instructions with multisensory support tools, using pictures, handouts, and props, repeating key parts	• Giving clear instructions
		• Seating S in a place where there are fewer stimuli	• Environmental school or study settings
	• Supporting S’s pre-academic skills, focusing on executive functioning skills		
		• Small chunks and predictable class organization	
		• Document managing skills: homework and note-taking support, while motivating the adolescent S	
Session 4	• Ignoring: waiting to praise	• Not reacting to S’s less-adaptive behaviors	• Using ignoring skills and focusing on positive points
		• Focusing on S’s positive aspects, and the effect of maximizing adolescent S’s strengths	
Session 5	• Setting limitations: dealing with S’s panic and emotional problems	• Making contracts	• Using limitation setting skills
		• Preparing a space to relax and dealing with S’s emotional control issues and sensory problems	
		• Reflecting on S’s changes in major issues from intake interviews	
	• Reflection		

S = Student.

**Table 5:** Descriptive statistics for student and teacher outcome measures and results of ANCOVA.

			**Time 1**		**Time 2**				
									Partial
Measure	Group	*n*	M	SD	M	SD	F(1, 27)	*p*	*η* ^2^
**TRF**									
Total	IT	13	67.38	23.94	57.46	24.38	11.04	0.003 *	0.29
	DTC	17	58.06	24.65	63.47	24.86			
Internalization	IT	13	14.69	11.23	12.54	9.72	3.97	0.056	0.13
	DTC	17	11.12	7.92	12.71	8.28			
Externalization	IT	13	15.85	13.94	12.54	8.98	‡		
	DTC	17	14.41	13.71	15.71	13.13			
**Subscales:**									
Withdrawal	IT	13	4.62	3.10	3.54	3.21	2.73	0.110	0.09
	DTC	17	3.65	2.76	4.18	2.51			
Somatic problems	IT	13	1.00	1.47	1.31	1.38	‡		
	DTC	17	0.94	1.60	1.12	1.73			
Anxiety	IT	13	9.69	8.67	8.31	8.07	3.23	0.084	0.11
	DTC	17	7.00	5.95	8.00	6.50			
Social problems	IT	13	9.62	5.19	8.31	5.50	7.40	0.011 *	0.22
	DTC	17	7.18	3.24	7.88	3.87			
Thought problems	IT	13	3.00	2.80	2.62	2.43	‡		
	DTC	17	2.00	2.87	2.12	2.80			
Attention problems	IT	13	18.08	6.56	16.46	7.18	5.07	0.033 *	0.16
	DTC	17	17.88	7.63	19.06	8.39			
Delinquent behavior	IT	13	3.23	3.22	2.46	2.76	6.55	0.016 *	0.20
	DTC	17	3.29	3.18	3.88	3.22			
Aggressiveness	IT	13	12.62	11.23	10.08	7.01	‡		
	DTC	17	11.12	11.13	11.94	10.44			
Other problems	IT	13	5.54	2.37	4.38	2.50	‡		
	DTC	17	5.00	2.98	5.29	2.62			
			**Time 1**		**Time 2**				
									Partial
Measure	Group	*n*	M	SD	M	SD	F(1, 27)	*p*	*η* ^2^
**SRS 2**									
Total	IT	13	93.77	20.90	88.08	24.90	4.22	0.050	0.14
	DTC	17	80.82	23.11	86.41	24.94			
SCI (Social Communication and Interaction)	IT	13	77.00	16.58	74.08	20.09	2.59	0.119	0.09
	DTC	17	66.82	19.05	71.59	20.68			
RRB (Restricted interests and Repetitive Behavior)	IT	13	16.77	7.66	14.00	6.48	4.38	0.046 *	0.14
	DTC	17	14.06	6.23	14.82	6.60			
**Subscales:**									
Awareness	IT	13	10.23	3.17	10.69	3.47	‡		
	DTC	17	11.76	3.61	12.47	2.81			
Cognition	IT	13	17.08	3.45	16.08	4.05	2.91	0.100	0.10
	DTC	17	15.47	5.28	16.94	5.20			
Communication	IT	13	34.92	8.89	34.00	11.98	‡		
	DTC	17	28.47	8.23	29.76	10.18			
Motivation	IT	13	14.77	4.48	13.31	5.31	‡		
	DTC	17	11.12	4.81	12.41	4.99			
			**Time 1**		**Time 2**				
									Partial
Measure	Group	*n*	M	SD	M	SD	F(1, 27)	*p*	*η* ^2^
**CDQ for Teachers**									
Q1: Waiting for S’s growth	IT	13	4.08	0.76	4.31	0.63	7.60	0.010 *	0.22
	DTC	17	3.76	0.90	3.47	0.87			
Q2: Accepting S’s developmental difficulties	IT	13	4.08	0.86	4.31	0.86	5.81	0.023 *	0.18
	DTC	17	4.00	0.87	3.65	0.79			
Q3: Having S do what (s)he can do by her/himself	IT	13	4.15	0.69	4.62	0.51	‡		
	DTC	17	3.41	1.23	3.41	1.06			
Q4: Praising S once or more a day	IT	13	3.31	1.03	3.92	0.95	3.87	0.060	0.13
	DTC	17	2.94	1.14	3.12	1.11			
Q5: Making a space for S to relax	IT	13	3.46	1.27	3.85	0.99	8.29	0.008 *	0.24
	DTC	17	3.24	0.97	2.82	1.07			
Q6: Helping S make friends	IT	13	3.54	1.20	3.77	0.93	‡		
	DTC	17	3.29	1.05	2.82	1.02			
Q7: Dealing with S’s maladaptive behaviors	IT	13	3.92	0.95	4.31	0.63	7.96	0.009 *	0.23
	DTC	17	3.29	1.05	3.24	0.97			
Q8: Providing appropriate support to S’s family	IT	13	3.23	1.24	3.69	1.18	4.03	0.055	0.13
	DTC	17	2.65	1.41	2.65	1.22			
Q9: Reducing the frequency of blaming myself or my career confidence	IT	13	3.92	1.12	4.15	0.90	2.64	0.116	0.09
	DTC	17	3.65	1.27	3.53	1.01			
Q10: Reducing my anxiety about S	IT	13	3.77	1.01	4.00	1.00	1.55	0.224	0.05
	DTC	17	3.06	0.97	3.24	0.97			
Q11: Spending my time for my health and pleasure	IT	13	4.08	0.86	4.31	0.86	‡		
	DTC	17	4.00	1.00	3.94	0.97			
Q12: Reducing troubles at school because of S’s behaviors	IT	13	3.46	0.78	4.23	0.60	12.63	0.001 *	0.32
	DTC	17	3.24	1.15	3.24	0.83			
Q13: Having other Ts support S	IT	13	4.00	1.08	4.08	0.95	0.76	0.391	0.03
	DTC	17	3.59	0.94	3.59	1.00			
Q14: Consulting with my coworkers	IT	13	4.54	0.52	4.31	0.75	‡		
	DTC	17	3.76	0.75	3.88	0.86			
Q15: Sharing my feelings and worries with other Ts	IT	13	4.23	0.93	4.08	0.95	0.27	0.608	0.01
	DTC	17	3.71	0.77	3.88	0.93			
Q16: Using medical and/or educational consultant organizations	IT	13	4.00	1.29	3.77	1.01	‡		
	DTC	17	3.35	1.22	3.18	1.24			
Q17: Understanding S’s behaviors and thoughts	IT	13	3.31	1.18	3.62	0.77	‡		
	DTC	17	3.29	0.99	3.41	0.80			
Q18: Feeling happy and having fun with S	IT	13	3.38	1.19	3.77	1.09	0.33	0.568	0.01
	DTC	17	3.53	1.07	3.71	1.16			

M = mean. SD = standard deviation. TRF: Teacher’s Report Form of the Child Behavior Checklist. SRS-2: Social Responsiveness Scale-2. SCI: social communication and interaction. RRB: restricted interests and repetitive behavior. CDQ for Teachers: Confidence Degree Questionnaire for Teachers. T: teacher. S: student. ‡: assumptions for ANCOVA were not met; these data are not included in our analysis. * significant difference: *p* < 0.05.

**Table 6:** Semi-structured interview results of teachers at Time 2 (IT group, *n* = 13) and Time 3 (DTC group, *n* = 13; 1 missing data).

Content: Questions	(Total *n* = 26) %
General Satisfaction with TT program: Are you satisfied with the	
TT program?*―Yes*	88.46
Students’ Behavior Improvements: Did you find any changes to	
or improvements in your target student’s behaviors? *―Yes*	80.77
Percentage of improvements in students’ behavior modification	
on each major issue at Time 1 (Table 1)	
(1) Attitude toward studies and pre-academic skills	75.00
(2) Behaviors toward teachers	91.67
(3) Behaviors toward peers	30.77
(4) Emotional control	60.00
(5) Following the teacher’s instructions	62.50
(6) Friendship with peers	12.50
(7) Rule-breaking	71.43
(8) Reducing impulsive or hyperactive behaviors	60.00
(9) Others	25.00
Changes in Teachers’ Cognition or Attitude: Did you find any	
changes in your cognition or attitude toward the target student?*―Yes*	92.31
